# Long noncoding RNA *MIR31HG* and its splice variants regulate proliferation and migration: prognostic implications for muscle invasive bladder cancer

**DOI:** 10.1186/s13046-020-01795-5

**Published:** 2020-12-17

**Authors:** Sheng Wu, Katja Nitschke, Thomas Stefan Worst, Alexander Fierek, Cleo-Aron Weis, Markus Eckstein, Stefan Porubsky, Maximilian Kriegmair, Philipp Erben

**Affiliations:** 1 Department of Urology and Urosurgery, Medical Faculty Mannheim, University of Heidelberg, 68167 Mannheim, Germany; 2 Department of Oncology, The First Affiliated Hospital of Anhui Medical University, Hefei, 230032 Anhui China; 3 Institute of Pathology, Medical Faculty Mannheim, University of Heidelberg, 68167 Mannheim, Germany; 4 Institute of Pathology, University Hospital Erlangen, Friedrich-Alexander-University Erlangen-Nürnberg, 91052 Erlangen, Germany

**Keywords:** LncRNA, *MIR31HG*, Muscle invasive bladder cancer, Biomarker, Molecular subtype

## Abstract

**Background:**

Growing evidence supports the pivotal role of long non-coding RNAs (lncRNAs) in the regulation of cancer development and progression. Their expression patterns and biological function in muscle invasive bladder cancer (MIBC) remain elusive.

**Methods:**

Transcript levels of lncRNA miR-31 host gene (*MIR31HG*) and its splice variants were measured in our MIBC cohort (*n* = 102) by qRT-PCR, and validated in silico by the TCGA cohort (*n* = 370). Kaplan-Meier and multiple Cox regression analysis were conducted to evaluate the survival significance of *MIR31HG* and its splice variants. Functional experiments were performed to examine the proliferation and migration abilities of *MIR31HG* and its splice variants by knockdown approaches.

**Results:**

In this study, a decreased expression of *MIR31HG* was found in bladder cancer cells and tissues, except in the basal subtype. Survival analysis showed that high expression of *MIR31HG* was associated with poor overall survival (OS) and disease-free survival (DFS) in patients with MIBC of basal subtype. Two splice variants of *MIR31HG* lacking exon 1 (*MIR31HGΔE1*) and exon 3 (*MIR31HGΔE3*) were identified to have specific expression patterns in different molecular subtypes of our MIBC cohort. *MIR31HGΔE3* was highly expressed in basal subtype tumors. A high expression of *MIR31HGΔE1* and *MIR31HGΔE3* was associated with worse OS and DFS in our cohort. In vitro experiments revealed that knockdown of *MIR31HG* inhibits cell proliferation, colony formation, and migration in bladder cancer. Cell proliferation and migration assays after knockdown of splice variants of *MIR31HG* showed corresponding roles for the full-length transcript.

**Conclusions:**

Our study demonstrates that *MIR31HG* and its splice variants could serve as biomarkers for the classification and prognosis prediction of patients with MIBC.

**Supplementary Information:**

The online version contains supplementary material available at 10.1186/s13046-020-01795-5.

## Background

Bladder urothelial carcinoma (BLCA) is the tenth most common cancer worldwide with an estimated 550,000 new cases and 200,000 deaths in 2018 [1]. It is widely accepted that BLCA is a heterogeneous disease, which is classified into two distinct subtypes: non-muscle invasive bladder cancer (NMIBC) and muscle invasive bladder cancer (MIBC) [2]. MIBC is responsible for most cases involving metastases leading to death, which arise from 10 to 20% of advanced NMIBC cases, and its clinical management remains limited [3]. Currently, several classifications have proposed sets of molecular classes, including basal and luminal subtypes, which are partially characterized by *KRT5* and *KRT20* gene expression [4]. In addition, epidermal growth factor receptor (EGFR) is expressed at high level in basal tumors, involved in controlling the basal gene signature [4]. Although a number of groups have reported molecular classifications of BLCA to evaluate the severity and prognosis of this disease, reliable and effective biomarkers for early diagnosis and prognostic prediction are still lacking [5, 6]. Thus, in-depth understanding of molecular events and underlying mechanisms involved in the carcinogenesis of MIBC may provide effective therapeutic targets and predictive biomarkers, which are urgently needed.

Long noncoding RNAs (lncRNAs) are a class of RNA transcripts, which are longer than 200 nucleotides and do not have protein-coding capacity [7]. Recent studies have demonstrated that lncRNAs play essential roles in a wide range of biological processes, such as proliferation, apoptosis, cell cycle arrest, cell migration, and invasion [8, 9]. Furthermore, it has been shown that several lncRNAs are deregulated in many tumors and may be involved in both carcinogenesis and cancer metastasis [10]. Recently, the miR-31 host gene (*MIR31HG*, also known as *LOC554202*) has been identified in several cancers, such as breast, colorectal, gastric cancer, and pancreatic ductal adenocarcinoma [11–14]. It is also reported that *MIR31HG* expression was down-regulated in BLCA cell lines and tumor tissues [15]. However, the functional role of *MIR31HG* and its association with molecular classifications in BLCA are as yet unknown.

Despite the continuously growing knowledge on lncRNAs and cancer, a reliable clinical molecular marker has not yet been found. Considering that dysregulation of mRNA splicing can trigger cancer signaling pathways and contribute to almost all hallmarks of cancer [16], the alternative splicing of lncRNAs may also impact cellular processes, which could open new possibilities for biomarker discovery. Previously, it was reported that splice variants of osteopontin have prognostic value in breast cancer [17]. In BLCA, CD44 splice variants have been demonstrated to be involved in tumor progression and chemosensitivity [18]. In addition, the lncRNA *PVT1* and its splice variant are highly expressed in clear cell renal cell carcinoma, and function as oncogenic transcripts [19]. These findings advocate for the use of lncRNAs and their splice variants as tissue-specific transcripts and promising prognostic biomarkers in certain cancers. However, the precise function of most lncRNAs and the mechanisms of their molecular regulation remains to be elucidated.

In this study, the expression level and clinicopathologic significance of *MIR31HG* were first evaluated by in silico database analysis and then by qRT-PCR in a cohort from our institution. Upon knockdown of *MIR31HG*, a series of in vitro experiments was performed to investigate the effects of *MIR31HG* on proliferation, colony formation, and migration of BLCA cells. Transcript-specific knockdown with consecutive cell functional assays were performed to determine the role of *MIR31HG* splice variants and to investigate their association with molecular subtypes of MIBC. Further survival analyses were carried out to determine if *MIR31HG* and its splice variants could be used as prognostic biomarkers.

## Materials and methods

### Patients and tissue samples

This study retrospectively enrolled 102 patients who received radical cystectomy (RC) at the Department of Urology and Urosurgery of the University Medical Centre Mannheim, between 2008 and 2012, and who had a histological diagnosis of MIBC (males: *n* = 74, 73%, median age: 71 years, range: 41–88 years; females: *n* = 28, 27%, median age: 73 years, range: 47–86 years; Mannheim cohort). All patients were treated with RC and bilateral lymphadenectomy without preoperative chemotherapy or radiotherapy. With the help of the clinic’s internal documentation program, the following parameters were collected after examination of the pathology findings: sex, age, T-stage, N-stage, M-stage, grading, lymphovascular invasion (LVI), blood vessel invasion (VI), simultaneous carcinoma in situ (CIS), multifocality, and soft tissue positive surgical margin.

Formalin fixed paraffin embedded (FFPE) tumor tissue samples were evaluated for pathological stage according to the 2017 TNM classification from the Union for International Cancer Control (UICC) [20]. Tumors were graded using the 2017 WHO/ISUP classification [21]. Studies involving human participants were approved by the ethical board of the University of Heidelberg (2015-549 N-MA) and performed in accordance with relevant guidelines and regulations. The Cancer Genome Atlas cohort (TCGA, Provisional) contained RNA sequencing data of 407 patients with MIBC and complete clinicopathological and follow-up data (males: *n* = 300, 73.7%, median age: 68 years, range: 34–90 years; females: *n* = 107, 26.3%, median age: 72 years, range: 43–90 years; TCGA cohort). Thirty-seven patients with tumor stage T1 or not defined T stage were excluded from the survival analysis (*n* = 370).

### Database

Expression data in transcripts per million (TPM) of 25 human bladder cancer cell lines were collected from the Cancer Cell Line Encyclopedia [22]. Expression of *MIR31HG* was analyzed by Expression Atlas (https://www.ebi.ac.uk/gxa/home) and normalized by TPM. Transcript expression data of 370 BLCA samples and 21 normal samples were collected from TCGA (https://tcga-data.nci.nih.gov/tcga/) and analyzed by cBioPortal (http://www.cbioportal.org/), which normalized the samples by reads per kilobase million (RPKM) and log2 transformation. Expression of transcripts, exons and junctions was collected and analyzed using the Xena online exploration tool (https://xena.ucsc.edu/) and TSVdb (http://www.tsvdb.com) [23]. The lncRNA-protein interactions were analyzed by lncPro [24]. lncPro yields a score using amino acid and nucleotide sequences. This score can be used to measure the interaction between a pair of lncRNA and protein. The sequence of *MIR31HG* and protein sequences were obtained from the NCBI database (http://www.ncbi.nlm.nih.gov/).

### Cell culture

In this study six different cell lines were used, including a normal human urothelium cell line (UROtsa), a basal-like urothelial carcinoma cell line (SCaBER), two luminal-like urothelial carcinoma cell lines (RT112 and RT4), and two mixed-type urothelial carcinoma cell lines (UMUC3 and T24). UROtsa cells were cultured in Roswell Park Memorial Institute medium (RPMI) supplemented with 5% fetal bovine serum (FBS). RT112, RT4, SCaBER, and UMUC3 cells were cultured in Dulbecco’s modified Eagle’s medium (DMEM) containing 10% FBS. T24 cells were cultured in McCoy’s 5A medium containing 10% FBS. UMUC3, SCaBER, RT112, and T24 cells were obtained from the European Collection of Authenticated Cell Cultures (ECACC), RT4 from the American Type Culture Collection (ATCC), and UROtsa cells from a collaborator. Before starting the experiments, all cell lines were authenticated by Multiplexion (Heidelberg, Germany).

### siRNA transfection

A pool of four different small interfering RNAs (siRNAs) against *MIR31HG* (designated si-*MIR31HG*, set of 4), and transcript-specific siRNAs against *MIR31HGΔE1* and *MIR31HGΔE3* were transfected into urothelial cells using the DharmaFECT1 siRNA transfection reagent (GE Healthcare Dharmacon, Inc., CO, USA). Scrambled siRNA was used as negative control. All sequences of the siRNAs are listed in Table S1.

### Cell viability assay

Cell viability was analyzed using the 3-(4,5-dimethylthiazol-2-yl)-5-(3-carboxymethoxyphenyl)-2-(4-sulfophenyl)-2H-tetrazolium (MTS) assay (Promega, Wis, USA) according to the manufacturer’s instructions with six replicates. MTS solution was added at 0, 24, 48, and 72 h after different siRNA transfections and the absorbance was measured using a microplate reader at 492 nm.

### Colony formation assay

For colony formation assays, urothelial cells were seeded in six-well plates at a concentration of 5000 cells per well in triplicates, and cultured for 7 days before staining viable colonies with crystal violet (Sigma-Aldrich, Darmstadt, Germany). The staining intensity of the colonies was quantitated using ImageJ software [25].

### Migration assay

Cell migration was evaluated by a wound-healing assay. Cells were seeded in cell culture inserts (ibidi GmbH, Munich, Germany) and the open wound area was measured after 12 h. The proportion of the wound area was quantitated using TScratch software [26].

### RNA extraction and qRT-PCR

Total RNA of cells was isolated using RNeasy Mini kits (Qiagen, Hilden, Germany) according to the manufacturer’s instructions. For FFPE tissues, RNA was extracted and enriched using the magnetic-bead-based XTRAKT FFPE Kit (Stratifyer, Cologne, Germany) according to the manufacturer’s instructions [27]. Next, reverse transcription was performed for cell samples using the M-MLV Reverse Transcriptase kit (Thermo Fisher Scientific, Waltham, MA, USA), and for FFPE samples using the Superscript III reverse transcriptase kit (Thermo Fisher Scientific, Waltham, MA, USA) with sequence-specific primers. qPCR was used to measure relative mRNA expression with TaqMan Fast Advanced Master Mix (Thermo Fisher Scientific, Waltham, MA, USA). β-Glucuronidase (GUS) and calmodulin2 (Calm2) were measured as reference genes [28]. The relative mRNA expression level was normalized to reference genes and determined using the 40-∆CT or 2^-∆∆CT^ method for cell culture samples and FFPE samples, as previously described [29, 30]. All primers and probes used in this study are shown in Table S2.

### Statistical analysis

Statistical analyses were performed using SPSS 20.0 software (IBM, Chicago, IL, USA) and GraphPad Prism 6.0 (GraphPad Software, La Jolla, CA, USA). A Kolmogorov–Smirnov (K-S) test was used to determine whether the data were normally distributed. Student’s t-tests were used to compare groups of normally distributed numerical data, while the Mann–Whitney U and Kruskal–Wallis tests were used to compare non-normally distributed numerical data. Linear regression was used to determine the efficiency of amplification. Spearman tests were used to test the correlation between different gene expressions. The cut-off values of the high and low *MIR31HG* expression groups were determined by receiver operating characteristic (ROC) curve analysis in the Mannheim cohort [31]. Similarly, in the TCGA cohort, the cut-off value (log2 value) of high and low *MIR31HG* expression groups were determined by ROC curve analysis. The Cox regression model was used for univariable and multivariable analysis to calculate hazard ratio (HR). For multivariable analysis, parameters with a cut-off value of *p* < 0.2 inform the univariable analysis were included. The expression of *KRT5* was used as a marker for basal phenotype. High *KRT5* and low *KRT5* were defined as expression above and below median. Survival rates of patients were calculated using the Kaplan–Meier method, and comparisons were made with the log-rank test. In all cases, *p* < 0.05 was considered statistically significant.

## Results

### MIR31HG expression was subtype-specific in bladder cancer tissues and cell lines

**Fig. 1:**
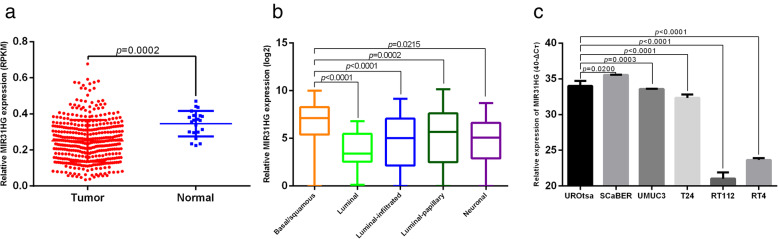
Expression of *MIR31HG* in BLCA tissue samples and cell lines. **a** In the TCGA cohort data, *MIR31HG* was down-regulated (median expression 0.2435) in tumors compared with normal tissues (median expression 0.3549). **b** In the TCGA cohort, expression of *MIR31HG* was higher in basal/squamous (median expression 7.10 with range of 0 to 9.96) subtype compared to luminal (median expression 3.16 with range of 0.13 to 6.8), luminal-infiltrated (median expression 4.99 with range of 0 to 9.16), luminal-papillary (median expression 5.53 with range of 0 to 9.92), and neuronal (median expression 4.92 with range of 0 to 8.7) subtypes. **c** In a normal urothelial cell line (UROtsa) and five BLCA cell lines (SCaBER, UMUC3, T24, RT112 and RT4), expression of *MIR31HG* was detected by qPCR. *MIR31HG* expression was significantly lower in UMUC3 and T24 cells, and higher in SCaBER cells compared to UROtsa cells. The lowest *MIR31HG* expression was observed in RT112 and RT4 cells

First, in silico analyses of expression of MIR31HG were performed. In the TCGA dataset, the expression of *MIR31HG* was down-regulated in MIBC (median expression 0.2435, *n* = 370) compared to normal tissues (median expression 0.3549, *n* = 23, *p* = 0.0002, Fig. 1a). Patients with BLCA in the TCGA cohort were classified into basal/squamous, luminal, luminal-infiltrated, luminal-papillary and neuronal subtypes according to mRNA clustering [32]. Among all subtypes, expression of *MIR31HG* in basal/squamous (median expression 7.10 with range of 0 to 9.96) subtype was the most abundant, which was higher than in luminal (median expression 3.16 with range of 0.13 to 6.8, *p* < 0.0001), luminal-infiltrated (median expression 4.99 with range of 0 to 9.16, *p* < 0.0001), luminal-papillary (median expression 5.53 with range of 0 to 9.92, *p* = 0.0002), and neuronal (median expression 4.92 with range of 0 to 8.7, *p* = 0.0215) subtypes (Fig. 1b). By clustering according to a trichotomous molecular classifications based on an alternative mRNA clustering of the TCGA cohort [33], it is also determined that the expression of *MIR31HG* was higher in the basal subtype (median expression 7.10 with range of 0 to 9.96) compared to the luminal (median expression 5.21 with range of 0 to 10.16) and infiltrated (median expression 4.99 with range of 0 to 9.16) subtypes (Fig. S1A). Furthermore, due to the significance of lymph node metastasis in MIBC cohort, the expression of MIR31HG in different lymph node status was analyzed. No significant difference was found between lymph node metastasis negative and positive groups (*p* = 0.2650, Fig. S1B).

Expression of *MIR31HG* was also detected in a normal urothelial cell line (UROtsa) and three BLCA cell lines (SCaBER, UMUC3 and T24) by qRT-PCR. Significantly lower expression of *MIR31HG* was observed in UMUC3 (mean expression 33.59, *p* = 0.0003) and T24 cells (mean expression 32.34, *p* < 0.0001) compared with UROtsa cells (mean expression 34.01). In contrast, expression of *MIR31HG* was higher in SCaBER cells (mean expression 35.53, *p* = 0.0200) compared to UROtsa cells. The lowest level of *MIR31HG* expression was observed in RT112 (mean expression 21.03, *p* < 0.0001) and RT4 cells (mean expression 23.64, *p* < 0.0001, Fig. 1c). Furthermore, *MIR31HG* expression was analyzed in RNA-seq data from the Cancer Cell Line Encyclopedia containing 25 BLCA cell lines, including 20 bladder urothelial cell carcinomas, a bladder squamous cell carcinoma, and four bladder carcinoma cell lines from unknown primaries. The data showed that *MIR31HG* expression levels in SCaBER cells (expression level: 10 TPM) were higher than in RT112 (expression level: 0 TPM), RT4 (expression level: 0 TPM), and UMUC3 cells (expression level: 3 TPM, Fig. S1C).

### Identification and expression of two splice variants of MIR31HG

Demographic and clinical-pathological data of the 102 patients with MIBC included in the Mannheim cohort are shown in Table 1. Median follow-up of the entire cohort was 21 months (range 3–121 months) and the median follow-up of surviving patients was 50 months (range 9–121 months). In total, 52 patients (50.98%) suffered a relapse (local relapse *n* = 6, lymph nodes and/or distant metastases *n* = 36, unclear metastasis pattern *n* = 10). Of the 56 (54.90%) patients who died during the follow-up, 38 (37.25%) of them died because of BLCA.

**Table 1:** Clinicopathological characteristics of patients and specimens of the Mannheim cohort

Clinicopathological Features	Number
Age	
< 70	43
≥70	59
Gender	
Male	74
Female	28
Stage	
T2	12
T3	69
T4	21
Lymph node metastasis	
Negative	70
Positive	24
Undefined	8

**Fig. 2:**
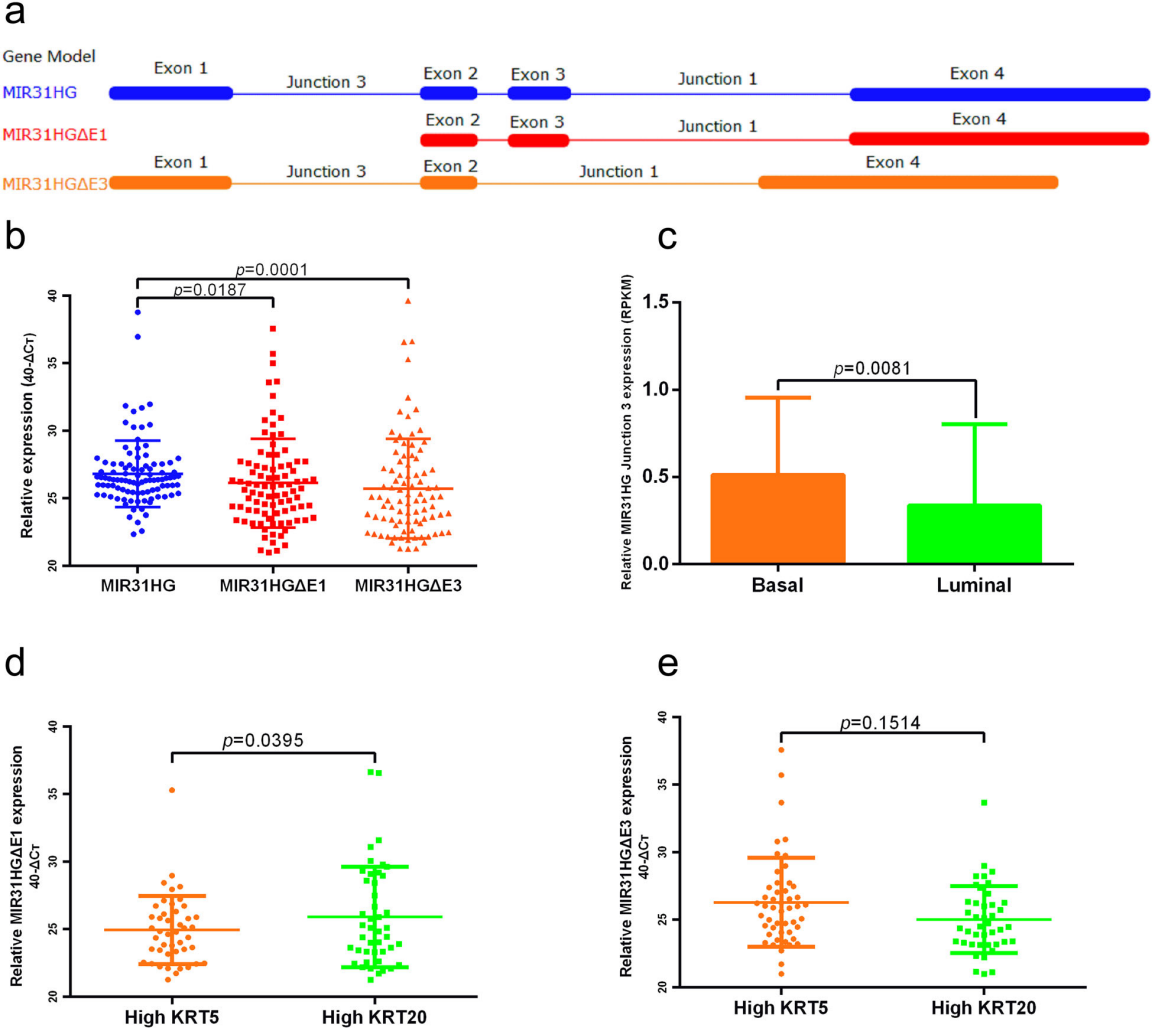
Expression of *MIR31HG* and two splice variants in the BLCA cohort. **a** Gene model of *MIR31HG* transcript and splice variants *MIR31HGΔE1* and *MIR31HGΔE3*. **b** In the Mannheim cohort, expression of *MIR31HG* (median expression 34.7) was significantly higher than in the splice variants, *MIR31HGΔE1* (median expression 35.5) and *MIR31HGΔE3* (median expression 36.7). **c** In the TCGA cohort, expression of *MIR31HGΔE3* was significantly higher in basal (median expression 0.6267) compared to luminal (median expression 0.3478) subtype. **d** In the Mannheim cohort, expression of *MIR31HGΔE1* was higher in the high *KRT20* group compared to the high *KRT5* group. **e** *MIR31HGΔE3* expression was higher in the high *KRT5* group compared to the high *KRT20* group in the Mannheim cohort

Four transcript variants of *MIR31HG* were retrieved from the National Center for Biotechnology Information (NCBI) nucleotide database. Transcript variant 1 (RefSeq ID: NR_027054.2) is the full transcript of *MIR31HG*, containing four exons and three junctions. For simplification, transcript variant 2 (RefSeq ID: NR_152877.1) lacking exon 1 was named *MIR31HGΔE1*, and transcript variant 4 (RefSeq ID: NR_152879.1) lacking exon 3 was named *MIR31HGΔE3*. Transcript-specific primers for each splice variant were designed and are listed in Supplementary Table 2. A model of the gene sequence of the *MIR31HG* transcript (ENST00000304425.3) and its two splice variants, *MIR31HGΔE1* and *MIR31HGΔE3*, is shown in Fig. 2a. In the Mannheim cohort, a significantly higher expression of *MIR31HG* (median expression 34.7) was found compared to the transcripts, *MIR31HGΔE1* (median expression 35.5, *p* = 0.0187) and *MIR31HGΔE3* (median expression 36.7, *p* = 0.0001, Fig. 2b). Expression of *MIR31HG* junction 3, which reflects *MIR31HGΔE3* expression, was significant higher in the basal (median expression 0.6267) compared to the luminal (median expression 0.3478) subtype in the TCGA cohort (*p* = 0.0081, Fig. 2c). As the expression of *KRT5* and *KRT20* can be used as a marker for basal and luminal phenotypes, respectively, the expression of *MIR31HG* was analyzed in terms of *KRT5* and *KRT20* dependent expression. High *KRT5* (Ct value > 36.55) and high *KRT20* (Ct value > 34.12) were defined as expression above median. *MIR31HGΔE1* expression was higher in the high *KRT20* group (*p* = 0.0395, Fig. 2d), and *MIR31HGΔE3* expression was higher in the high *KRT5* group (*p* = 0.1514, Fig. 2e).

### MIR31HG expression was associated with copy-number alterations

**Fig. 3:**
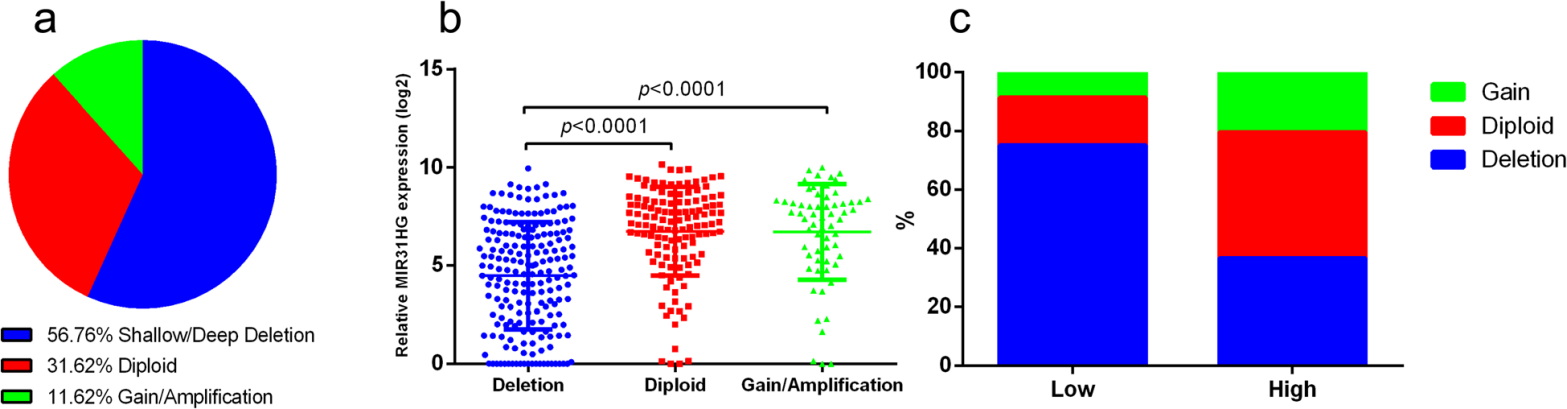
*MIR31HG* expression in association with copy-number alterations. **a** *MIR31HG* showed 56.76% of shallow/deep deletion, 31.62% of diploid and 11.62% of gain/amplification. **b** Significantly higher expression of *MIR31HG* was observed in the subgroup with gain (median expression 7.46) and diploid (median expression 7.12) compared to deletion (median expression 4.77). **c** High expression of *MIR31HG* was observed in the subgroup with deletion and diploid than in gain (n = 47, 20.44% in gain; n = 99, 43.04% in diploid; n = 84, 36.52% in deletion), while low expression was observed in the subgroup with deletion compared to diploid and gain (n = 15, 8.67% in gain; n = 28, 16.18% in diploid; n = 130, 75.15% in deletion)

The in silico analysis of *MIR31HG* copy-number alterations revealed a genetic alteration rate of 25% (92/370). Putative copy-number alterations including deep/shallow deletion, diploid, gain, and amplification were acquired from the GISTIC (Genomic Identification of Significant Targets in Cancer) algorithm. *MIR31HG* showed 56.76% of shallow/deep deletion (*n* = 210), 31.62% of diploid (*n* = 117), and 11.62% of gain/amplification (*n* = 43, Fig. 3a). The expression of *MIR31HG* was significantly higher in the subgroup with gain (median expression 7.46, *p* < 0.0001) and diploid (median expression 7.12, *p* < 0.0001) compared to those with a deletion alteration (median expression 4.77, Fig. 3b). High expression of *MIR31HG* was found in the subgroup with deletion and diploid alteration than in gain (*n* = 47, 20.44% in gain; *n* = 99, 43.04% in diploid; *n* = 84, 36.52% in deletion), while low expression was observed in the subgroup with deletion alteration, compared to diploid and gain (*n* = 15, 8.67% with gain; *n* = 28, 16.18% with diploid; *n* = 130, 75.15% with deletion, Fig. 3c).

### MIR31HG as prognostic marker for patients with basal subtype

**Fig. 4:**
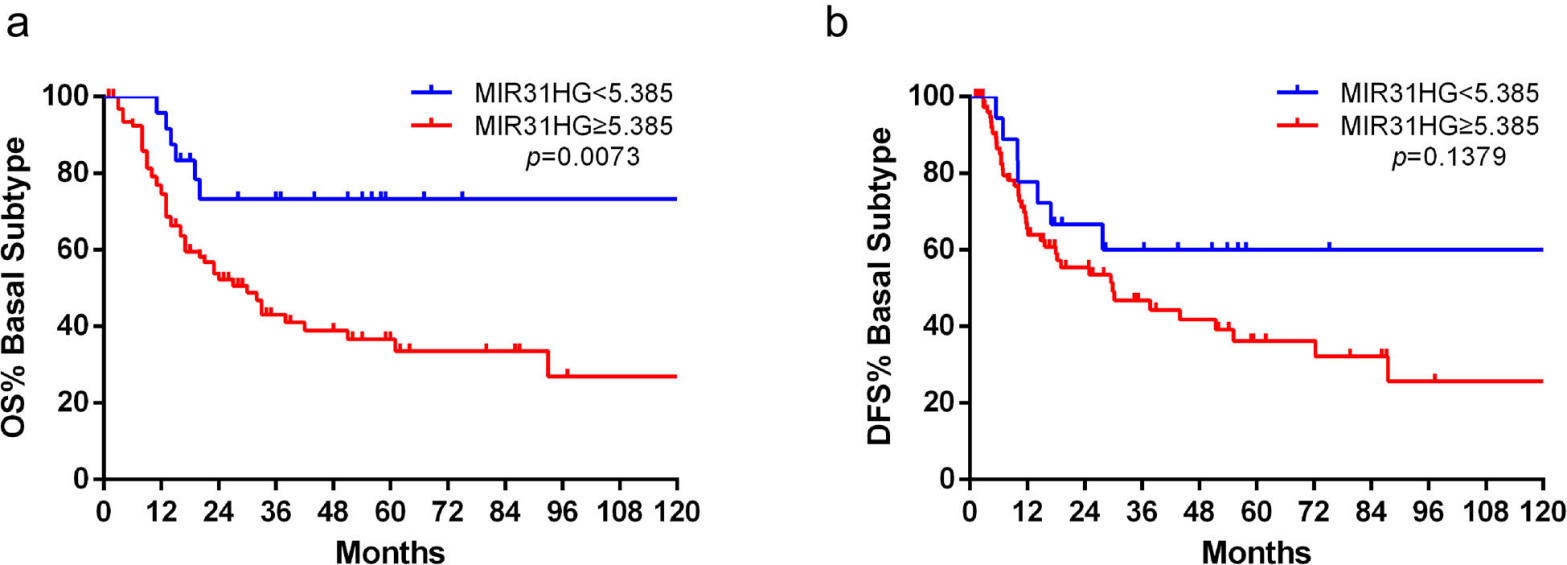
Kaplan-Meier plot of overall survival (OS) and disease free survival (DFS) were associated with *MIR31HG* risk stratification. **a** In the TCGA cohort with basal subtype, the group with high *MIR31HG* expression showed a worse OS compared to the group with low expression (median survival, 28 vs. 16 months, *p* = 0.0073). **b** In the TCGA cohort with basal subtype, the group with high *MIR31HG* expression showed a worse DFS compared to the group with low expression (median survival, 27 vs. 15 months, *p* = 0.1379). The numbers below the figures showed the number of patients at risk in each group

After the analysis of gene expression and copy-number alterations of *MIR31HG*, the association with clinical outcome and parameters was analyzed in the TCGA and the Mannheim cohort. The expression of MIR31HG showed no significant correlation with overall survival (OS) and disease free survival (DFS) in the full TCGA cohort (Fig. S2). Interestingly, survival analysis showed that patients of the TCGA cohort with basal subtype were significantly associated with OS and DFS based on *MIR31HG* risk stratification. The group with high *MIR31HG* expression showed a worse OS compared to the group with low expression (median survival, 28 vs. 16 months, *p* = 0.0073, Fig. 4a). Furthermore, the group with high *MIR31HG* expression showed a worse DFS compared to the group with low expression (median survival, 27 vs. 15 months, *p* = 0.1379, Fig. 4b).

### Splice variants of MIR31HG as prognostic markers for patients

**Fig. 5:**
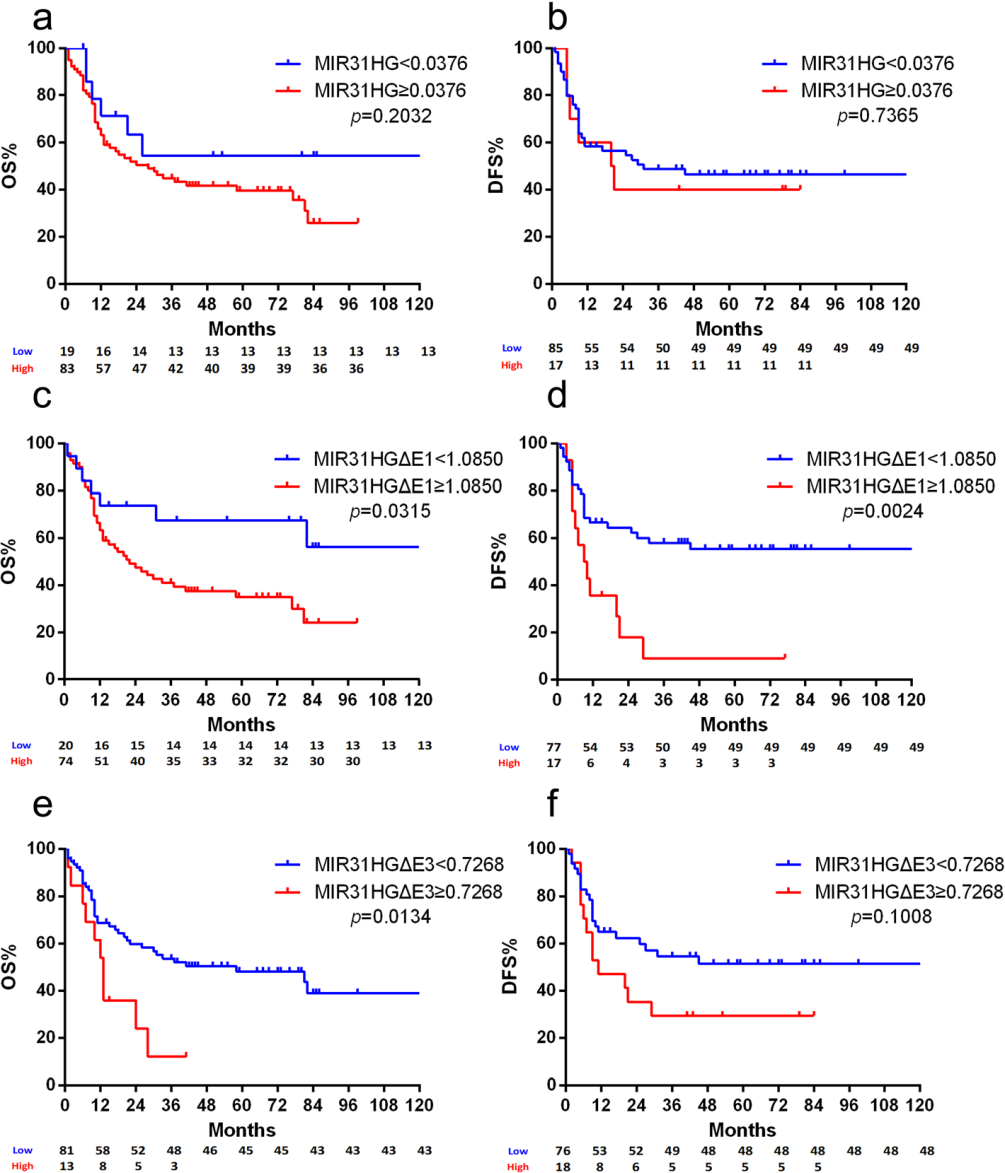
Kaplan-Meier plot of the Mannheim cohort of OS and DFS associated with *MIR31HG* and its splice variants risk stratification. No significant correlation was found with OS (**a**, median survival 18 vs. 21 months) and DFS (**b**, median survival 11 vs. 9 months for *MIR31HG*) in the group with full-length transcript of *MIR31HG*. The group with high *MIR31HGΔE1* expression showed a worse OS (**c**, median survival 15 vs. 38 months) and DFS (**d**, median survival 7 vs. 25 months) compared to the group with low expression. The group with high *MIR31HGΔE3* expression showed a worse OS (**e**, median survival 12 vs. 30 months) and DFS (F, median survival 9 vs. 15 months) compared to the group with low expression. The numbers below the figures showed the number of patients at risk in each group

In the Mannheim cohort, Kaplan-Meier analysis and the log-rank test were used to evaluate the association of the expression of two *MIR31HG* splice variants with OS and DFS. For the full-length transcript of *MIR31HG*, no significant difference in OS (median survival 18 vs. 21 months, *p* = 0.2032, Fig. 5a) and DFS (median survival 11 vs. 9 months for *MIR31HG*, *p* = 0.7365, Fig. 5b) was found between high and low expression levels. Tumors with both high *MIR31HGΔE1* (median survival 15 vs. 38 months, *p* = 0.0315, Fig. 5c) and *MIR31HGΔE3* expression (median survival 12 vs. 30 months, *p* = 0.0024, Fig. 5e) showed a worse OS. The groups with high *MIR31HGΔE1* (median survival 7 vs. 25 months, *p* = 0.0134, Fig. 5d) and *MIR31HGΔE3* (median survival 9 vs. 15 months, *p* = 0.1008, Fig. 5f) expression also showed a worse DFS. Tumors with high expression of *MIR31HG* Exon1–2 (Junction 3), which could partially present as *MIR31HGΔE3*, showed a worse OS than the group with low expression, in the TCGA cohort with basal subtype (median survival 15 vs. 17 months, *p* = 0.0298, Fig. S3A). No significant difference was found for DFS (median survival 12 vs. 15 months, *p* = 0.5670, Fig. S3B).

Similar to the analysis of *MIR31HG* in the TCGA cohort, the expression of *MIR31HG* and its splice variants were also analyzed regarding to OS and DFS in patients from Mannheim with basal subtype (high *KRT5* expression). No significant differences were found in OS (median survival 18 vs. 20 months, *p* = 0.6664, Fig. S4A) and DFS (median survival 17 vs. 15 months, *p* = 0.8690, Fig. S4B) between high and low expression of *MIR31HG*, also in high and low expression of *MIR31HGΔE1* (median survival 15 vs. 18 months for OS, *p* = 0.2338, Fig. S4C; median survival 29 vs. 17 months for DFS, *p* = 0.5984, Fig. S4D). Significant difference with OS was found between high and low expression of *MIR31HGΔE3* in the basal subtype cohort (median survival 13 vs. 22 months, *p* = 0.0194, Fig. S4E). Yet, no significant correlation was found with DFS (median survival 15 vs. 36 months, *p* = 0.3427, Fig. S4F).

In the univariable Cox regression analysis, *MIR31HGΔE1* (*p* = 0.0063) and *MIR31HGΔE3* (*p* = 0.0114) expression as well as the stage of cancer (*p* = 0.0092) were found to be predictive for patient outcome. In the multivariable Cox regression analysis, *MIR31HGΔE3* was identified as an independent prognostic factor (*p* = 0.0090), similar as stage (*p* = 0.0243). No significant differences of OS were found between basal (high *KRT5*) and non-basal (low *KRT5*) group in the univariable analysis. However, no significant correlation was observed between the full-length transcript of *MIR31HG* regarding to OS of the patients in the univariable analysis, nor with patient age, patient gender, lymph node status, and LVI (Table 2).

**Table 2:** Univariable and multivariable cox regression analysis of *MIR31HG* and its splice variants with clinicopathological features in the Mannheim cohort (*HR* hazard ratio, *CI* confidence interval, *LVI* lymphovascular invasion, *Basal* high *KRT5* expression, *Non-basal* low *KRT5* expression, significant *p* values are bold)

Factor	Univariable		Multivariable	
	HR (95% CI)	*p*	HR (95%CI)	*p*
**Diagnosis Age**				
<70 vs. ≥70	0.701(0.406–1.212)	0.2041	–	–
**Gender**				
Male vs. Female	0.980(0.535–1.795)	0.9485	–	–
**Stage**				
T2 vs. T3/4	0.401(0.202–0.797)	**0.0092**	0.445(0.220–0.900)	**0.0243**
**LVI**				
Negative vs. Positive	0.550(0.075–4.050)	0.5570	–	–
**Lymphnode Status**				
Negative vs. Positive	0.547(0.245–1.221)	0.1418	0.694(0.243–1.987)	0.4961
**Molecular Subtype**				
Basal vs. Non-basal	1.202(0.596–2.422)	0.6073	–	–
***MIR31HG***				
Low vs. High	1.720(0.681–4.347)	0.2522	–	–
***MIR31HGΔE1***				
Low vs. High	3.753(1.457–9.664)	**0.0063**	1.896(0.831–4.329)	0.1294
***MIR31HGΔE3***				
Low vs. High	2.522(1.235–5.149)	**0.0114**	2.805(1.293–6.083)	**0.0090**

### MIR31HG is required for BLCA tumorigenesis

**Fig. 6:**
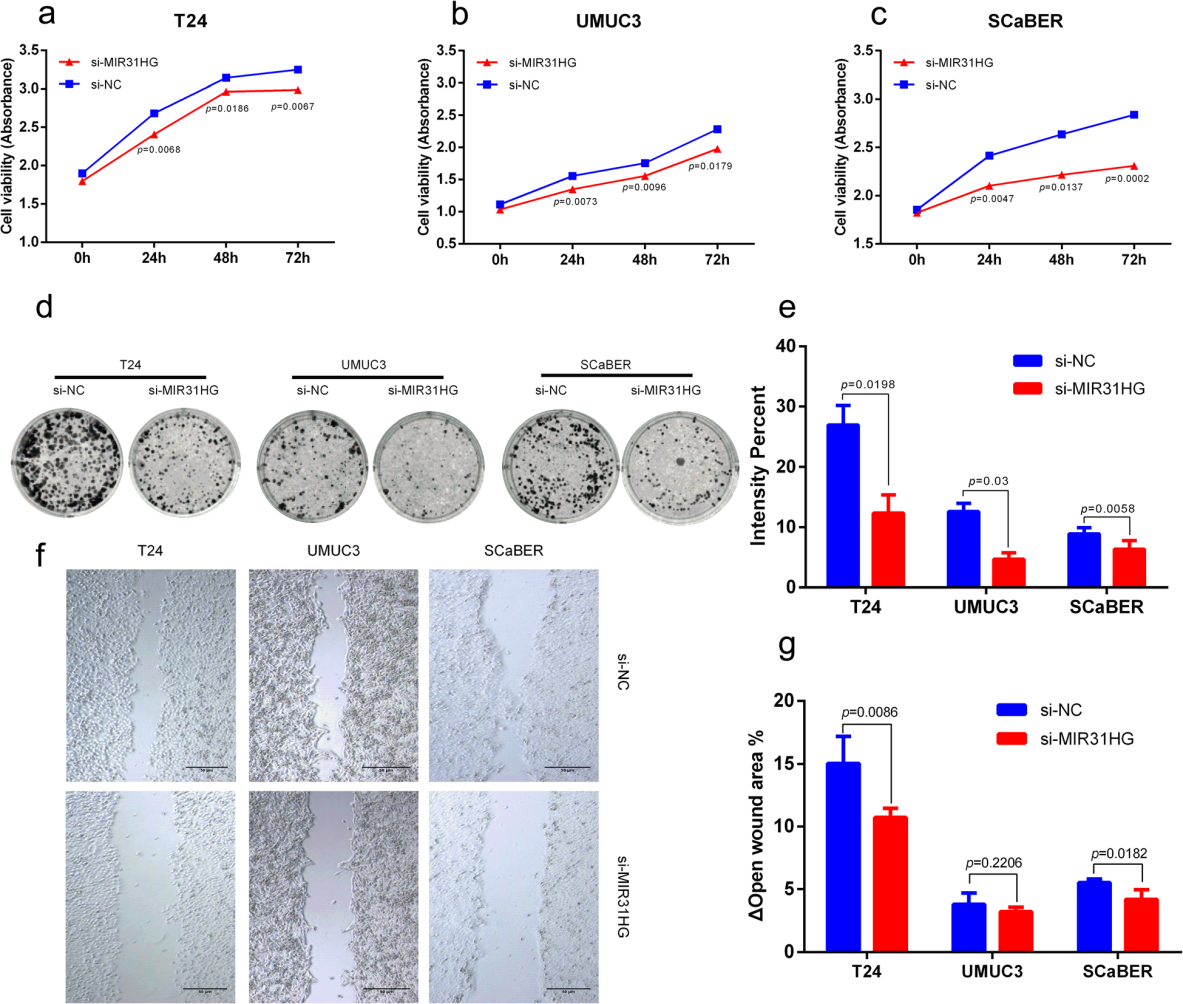
*MIR31HG* is required for BLCA tumorigenesis. **a**-**c** Cell viability was quantified using the MTS assay. T24 (A), UMUC3 (**b**) and SCaBER (**c**) cells with *MIR31HG* knockdown by siRNA showed lower cellular viability compared with control group with scramble siRNA (si-NC). **d** Colony formation was detected by measuring the stained cells after 7 days. T24, UMUC3, and SCaBER cells with *MIR31HG* knockdown showed fewer cell colonies compared to control group. **e** Staining intensity percentage of colony formation assay was analyzed by ImageJ software. BLCA cells with *MIR31HG* knockdown showed decreased staining intensity compared with control group. **f** Wound healing assay was measured using cell culture inserts. T24, UMUC3, and SCaBER cells with *MIR31HG* knockdown showed more open wound area compared with control group after 12 h. **g** Open wound area was quantified by calculating the percentage of change in open wound area after 12 h. BLCA cells with *MIR31HG* knockdown showed fewer changes in open wound area compared to the control group

To evaluate the possible role of *MIR31HG* in BLCA, a pool of four siRNAs against *MIR31HG* (designated si-*MIR31HG*, set of 4) were transfected in T24, UMUC3, and SCaBER cells. Each transfection resulted in a sufficient knocked down of *MIR31HG* expression. Cell viability was determined using the MTS assay based on absorbance at 490 nm after 0, 24, 48, and 72 h. T24, UMUC3, and SCaBER cells with *MIR31HG* knockdown by siRNA showed lower cellular viability at three time points compared to the control group transfected with scramble siRNA (si-NC, Fig. 6a-c). Moreover, colony formation was detected by culturing cells in a 6-well plate and stained with crystal violet after 7 days. T24, UMUC3, and SCaBER cells with *MIR31HG* knockdown showed fewer cell colonies compared to the control group (Fig. 6d). The staining intensity of the colony formation assay was quantified by calculating the colony intensity percentage. BLCA cells with *MIR31HG* knockdown showed a reduced formation of colonies compared to the control group (Fig. 6e). Migration was detected by a wound-healing assay. T24, UMUC3, and SCaBER cells with *MIR31HG* knockdown showed a larger open wound area compared with the control group after 12 h (Fig. 6f). The difference in open wound area after 12 h was quantified by calculating the percentage of change in the open wound area (open wound area at 12 h - open wound area at 0 h). BLCA cells with *MIR31HG* knockdown showed fewer changes in open wound area compared with the control group (Fig. 6g).

### Two splice variants of MIR31HG regulate BLCA growth with cell specificity

**Fig. 7:**
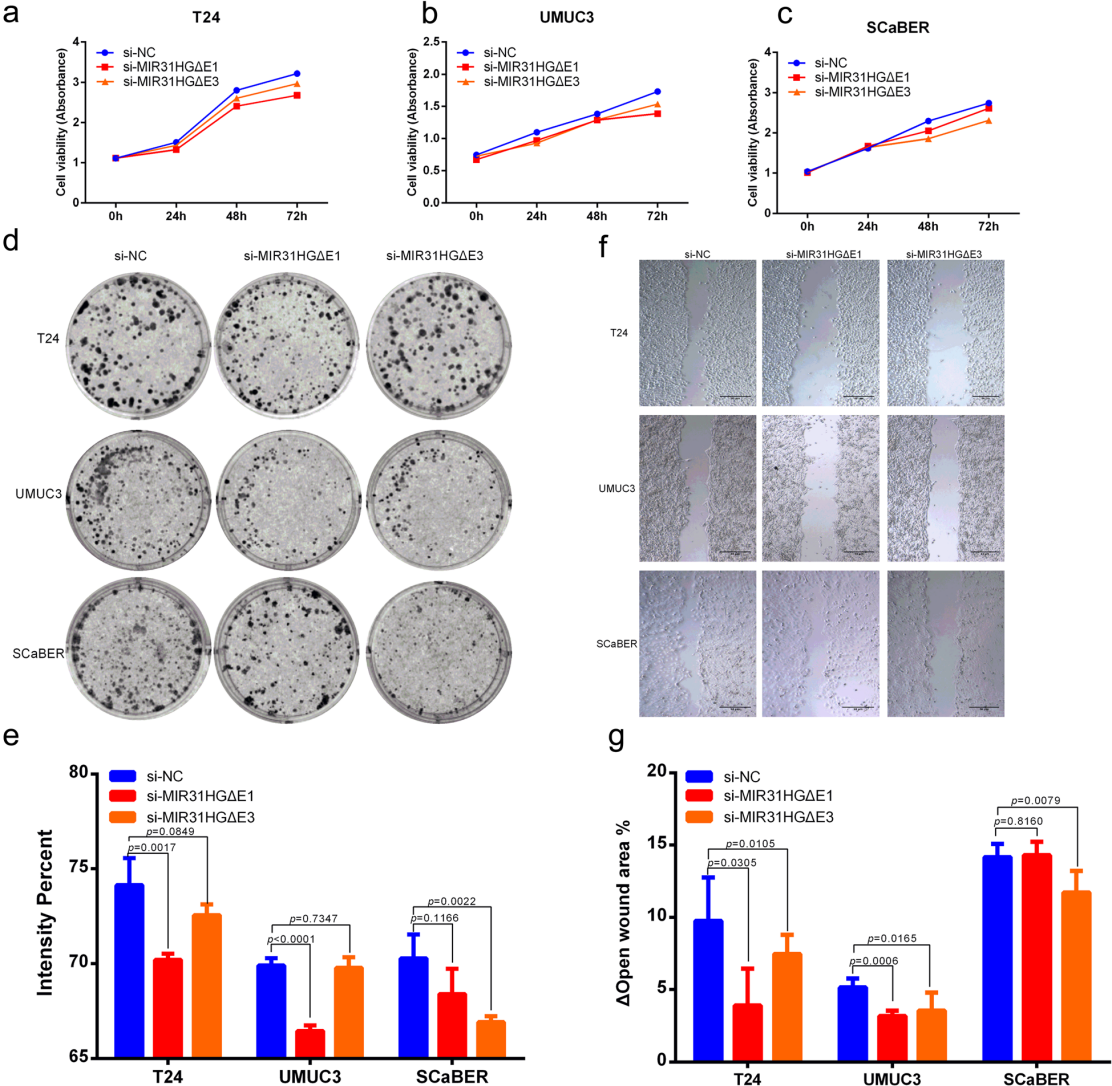
Two splice variants of *MIR31HG* are required for BLCA tumorigenesis with cell specificity. (A-C) Cell viability was quantified using the MTS assay. *MIR31HGΔE1* knockdown showed a significant decrease in absorbance in T24 (*p* = 0.0018, **a** and UMUC3 (*p* = 0.0029, **b** cells, and *MIR31HGΔE3* knockdown showed a significant decrease in absorbance in SCaBER (*p* = 0.0346, **c** cells after 72 h. **d** Colony formation was detected by measuring cell staining after 7 days. T24, UMUC3, and SCaBER cells with *MIR31HGΔE1* and *MIR31HGΔE3* knockdown by specific siRNA showed fewer cell colonies with cell specificity. **e** Staining intensity percentage of colony formation assay was analyzed by ImageJ sofware. T24 and UMUC3 cells with *MIR31HGΔE1* knockdown and SCaBER cells with *MIR31HGΔE3* knockdown showed a significant decrease in staining intensity compared to the control group. **f** Wound healing assay was measured using cell culture inserts. T24, UMUC3, and SCaBER cells with *MIR31HGΔE1* and *MIR31HGΔE3* knockdown by specific siRNA showed a larger open wound area compared with cell specificity after 12 h. **g** Difference in open wound area was quantified by calculating the percentage of change in open wound area after 12 h. T24 and UMUC3 cells showed fewer changes in the open wound area, in both *MIR31HGΔE1* and *MIR31HGΔE3* knockdown groups. SCaBER cells showed fewer changes in the open wound area in the *MIR31HGΔE3* knockdown group

To further evaluate the possible role of the transcripts *MIR31HGΔE1* and *MIR31HGΔE3* in BLCA, transcript-specific siRNAs were transfected into BLCA cells. Cell viability was quantified by MTS assay based on absorbance at 490 nm after 0, 24, 48, and 72 h. T24, UMUC3, and SCaBER cells with *MIR31HGΔE1* and *MIR31HGΔE3* knockdown by specific siRNA showed decreased cellular viability with cell specificity (Fig. 7a-c). *MIR31HGΔE1* knockdown showed significantly decreased absorbance in T24 (*p* = 0.0018, Fig. 7a) and UMUC3 (*p* = 0.0029, Fig. 7b) cells after 72 h, and *MIR31HGΔE3* knockdown resulted in a significant decrease in absorbance in SCaBER (*p* = 0.0346, Fig. 7c) cells exclusively after 72 h. Colony formation was detected by culturing cells in 6-well plates and staining with crystal violet after 7 days. T24, UMUC3, and SCaBER cells with *MIR31HGΔE1* and *MIR31HGΔE3* knockdown by specific siRNA showed fewer cell colonies with cell specificity (Fig. 7d). T24 and UMUC3 cells with *MIR31HGΔE1* knockdown showed a significantly reduced staining intensity compared with control transfected cells. SCaBER cells with *MIR31HGΔE3* knockdown showed significantly decreased staining intensity (Fig. 7e). Migration was detected with a wound healing assay using cell culture inserts. T24, UMUC3, and SCaBER cells with *MIR31HGΔE1* and *MIR31HGΔE3* knockdown by specific siRNA showed a larger open wound area compared with cell specificity after 12 h (Fig. 7f). The difference in open wound area after 12 h was quantified by calculating the percentage of change in open wound area (open wound area 12 h - open wound area 0 h). T24 and UMUC3 cells showed fewer changes in open wound area, both in the *MIR31HGΔE1* and *MIR31HGΔE3* knockdown groups. SCaBER cells showed fewer changes in the open wound area upon *MIR31HGΔE3* knockdown (Fig. 7g).

### *MIR31HG* is associated with EGFR pathway

To discover the potential mechanisms underlying the functions of *MIR31HG*, the related proteins were focused on EGFR, which was reported as a molecular signature of basal or squamous-like bladder cancer [34]. The lncRNA-protein interactions of *MIR31HG* and EGFR were analyzed by lncPro [24] and predicted 9 Isoforms of EGFR interacting. Besides EGFR and its isoforms, *MIR31HG* was predicted as interactive with phosphoinositide 3-kinase (PI3K) and receptor tyrosine-protein kinase erbB-2 (HER2) protein (score above 50, Table 3).

**Table 3:** Interaction scores of EGFR, PI3K HER2 protein and MIR31HG

Protein	Score
EGFR	72.4935
EGFR isoform a	75.0794
EGFR isoform b	93.4501
EGFR isoform c	83.9972
EGFR isoform d	93.8757
EGFR isoform e	72.5006
EGFR isoform f	75.7342
EGFR isoform g	73.3893
EGFR isoform h	82.7141
EGFR isoform i	84.5429
PI3K	75.5458
HER2 isoform a	70.9194
HER2 isoform b	82.7639
HER2 isoform c	79.0902
HER2 isoform d	69.6083
HER2 isoform e	91.4699

**Fig. 8:**
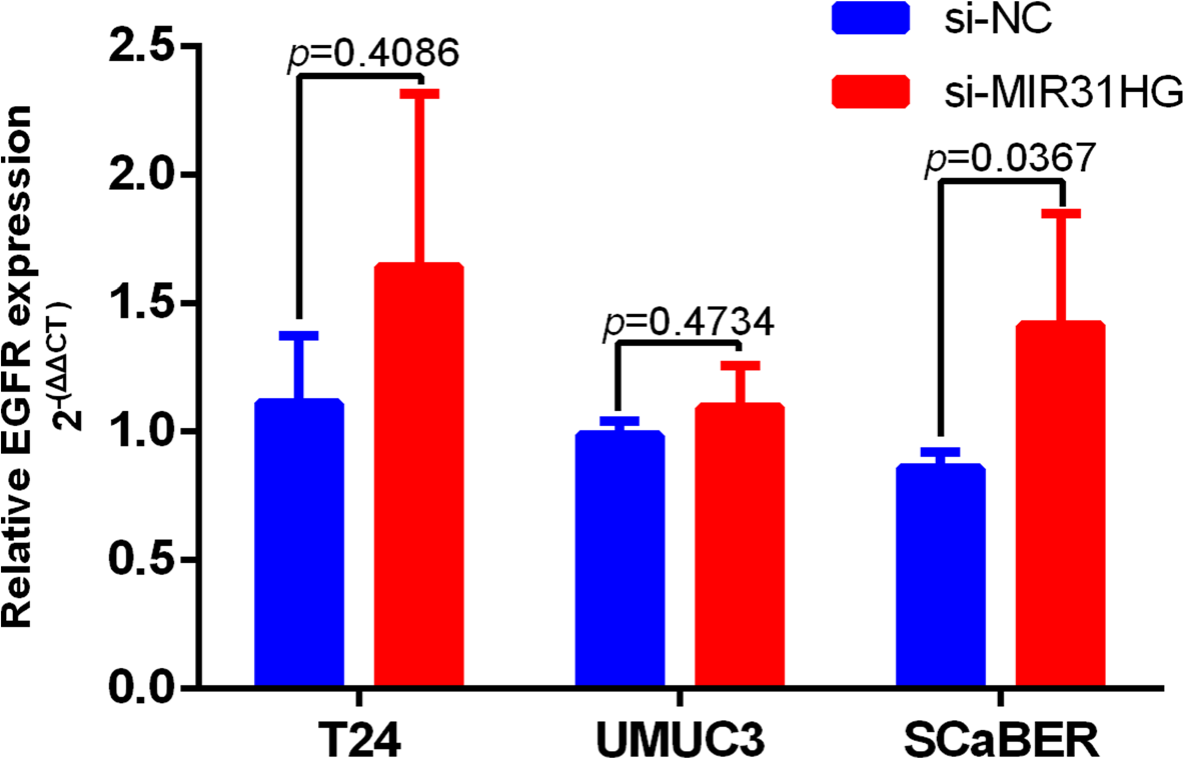
Expression of *EGFR* in BLCA cell lines with MIR31HG knockdown. To narrow the differences in *EGFR* expression between cell lines, the 2^−ΔΔCT^ values of expression data in T24, UMUC3 and SCaBER cells were normalized by the expression data in UROtsa. *EGFR* expression was significantly higher in SCaBER cells with *MIR31HG* knockdown compared to negative siRNA group. Expression of *EGFR* showed no significant difference between T24 and UMUC3 cells with MIR31HG knockdown and negative siRNA group

To further reveal the interaction with the coded gene, gene expression of *EGFR* was detected in *MIR31HG* knockdown cells. Significantly higher expression of *EGFR* was observed in SCaBER cells with *MIR31HG* knockdown compared to the control group (*p* = 0.0367, Fig. 8). No significant difference of expression was observed between T24 (*p* = 0.4086) and UMUC3 (*p* = 0.4734) cells with *MIR31HG* knockdown and control group (Fig. 8).

## Discussion

This study aimed to investigate the expression pattern and biological function of *MIR31HG* in MIBC, as well as its clinical significance and prognostic value in patients. In order to evaluate its role in tumorigenesis and progression, functional in vitro assays were combined with lncRNA expression analysis based on molecular subtypes and relevant clinicopathologic parameters.

In previous studies on multifarious tumors, *MIR31HG* showed a tissue-specific expression pattern. In breast cancer and non-small cell lung cancer (NSCLC) cells, *MIR31HG* expression was upregulated [11, 35]. In gastric cancer tissues and cell lines, *MIR31HG* was poorly expressed [13]. Another study showed that MIR31HG level is substantially upregulated in oral carcinoma, significantly associated with poor clinical outcomes and representing an independent prognostic predictor [36]. In our study, lower *MIR31HG* transcript levels were found in luminal-like and mixed-type BLCA cell lines compared with a normal urothelium cell line. Accordingly, down-regulated *MIR31HG* expression was found in cancer tissues compared to normal tissues, which supports expression results measured by qPCR from a previous study [15]. However, the previous results were measured in stage- and type-mixed BLCA tissues, and in this study, *MIR31HG* was measured in MIBC and associated with multiple molecular subtype respectively. In contrast, *MIR31HG* was found to be highly expressed in cells lines and clinical tumor samples with the basal subtype compared to luminal and other subtypes, indicating that *MIR31HG* not only shows tissue specific, but also subtype-specific overexpression in MIBC.

In contrast to a previous study, which reported that *miR-31* and *MIR31HG* are down-regulated in triple-negative breast cancer (TNBC) cell lines of basal subtype [37], the present study shows that *MIR31HG* is highly expressed in the BLCA cell line of basal subtype and markedly correlates with the survival of patients with MIBC basal subtype. This might be due to tissue specific expression of *MIR31HG*. In this study, two MIBC cohorts with multiple molecular subtypes were involved rather than single cell line, which may also lead to the dissimilar results. Further studies of BLCA preclinical models are needed for validation. It is noteworthy that two transcript variants of *MIR31HG* (*MIR31HGΔE1* and *MIR31HGΔE3*) were identified and their expression was analyzed in BLCA cells and MIBC tissues. Besides the different expression levels in MIBC patient tissues, the two splice variants showed distinguished expression patterns in basal and luminal subtypes, respectively. *MIR31HGΔE3* showed high expression in the basal subtype, both in the TCGA cohort and the Mannheim cohort, which is also observed for the group with high *KRT5* expression. In contrast, *MIR31HGΔE1* showed high expression in luminal subtype tumors in the Mannheim cohort, corresponding to tumors with a high *KRT20* expression.

Additionally, higher *MIR31HG* transcript levels were found to be associated with worse OS and DFS in the basal subtype cohort, but not in the whole TCGA cohort. It is the first time to discover the prognostic value of *MIR31HG* in BLCA, or associated with subtypes of tumors. Furthermore, expression of *MIR31HGΔE1* and *MIR31HGΔE3* was significantly associated with OS and DFS in the Mannheim cohort, rather than the full-length transcript of *MIR31HG*. In the TCGA cohort, it was demonstrated that *MIR31HGΔE3* expression was significantly associated with OS of the basal subtype group. These results, together with univariable and multivariable Cox regression analysis suggested that the alternative splice variants of *MIR31HG* may serve as potential biomarkers for certain molecular subtypes of MIBC, which could contribute to an individualized bladder cancer subclassification and therapy decision making. It is worth noting that lymph node status is not associated with OS based on survival analysis, which is controversial to other studies [38]. The possible reasons could be limited number of patients, diverse distribution of lymph node status and T stages as well as different scale of lymph node examination. Therefore, validation in larger patient cohorts with long-term follow-up is needed. In the Mannheim cohort, few patients were treated with neoadjuvant or adjuvant chemotherapy. For further validation, the number of positive lymph nodes and chemotherapy status should be also taken into consideration.

Two groups independently recognized the significance of a distinct basal MIBC subtype [39, 40]. According to classification of the TCGA, basal tumors were divided into two subsets that were largely distinguished by differential expression of biomarkers associated with EMT, which is a reversible developmental process by promoting invasion, metastasis, “stemness”, and drug resistance [32, 41]. The potential significance of the mesenchymal basal BLCA was identified using a “claudin-low” gene expression signature in breast cancer [42]. A previous study reported that tumor suppressor microRNA-361 was de-repressed by *MIR31HG* in osteosarcoma cells, leading to cell growth and mesenchymal phenotype [43]. These results may indicate that high expression *MIR31HG* could be served as a surrogate marker of poor outcome defined by relative activation of EMT and sponge of tumor suppressor. The discovery that *MIR31HG* is highly expressed and significantly outcome-correlated in MIBC with basal subtype, has complemented the selection of MIBC markers.

Due to its potential clinical relevance as a marker, the expression of *MIR31HG* and its underlying biological functions could be vital to the tumor. In this study, by knocking down *MIR31HG* expression using siRNA, diminished cell proliferation, colony formation, and migration were assessed in BLCA cell lines. This study is the first to highlight the function of *MIR31HG* in BLCA cells, which indicates that *MIR31HG* might serve as an oncogene in certain types of BLCA. With the in-depth study of MIR31HG, several downstream targets were found in present researches. For example, it is reported that overexpression of *MIR31HG* significantly decreased the expression of miR-575, enhanced the suppression of tumorigenicity 7 like (ST7L) in hepatocellular carcinoma (HCC). Thus, *MIR31HG* regulated *ST7L* expression through sponging miR-575, and acted as tumor suppressor in HCC [44]. Another published research showed that the level of *MIR31HG* in esophageal squamous cell carcinoma (ESCC) tissues was positively correlated with the expression of furin and matrix metalloproteinase 1 (MMP1). When *MIR31HG* was silenced, the expressions of furin and *MMP1* in ESCC cells were significantly inhibited. These results suggest that the involvement of *MIR31HG* in invasion and migration of ESCC cell may be partly achieved through the furin / *MMP1* pathway [45]. In a study in oral cancer, *MIR31HG* was identified as a hypoxia-inducible lncRNA and forms a complex with hypoxia-inducible factor-1 α (HIF-1α), thus as an adverse prognostic predictor for the cancer progression [36]. In addition, it was reported that *MIR31HG* could function as an oncogene that promotes pancreatic cancer progression, by acting as an endogenous sponge competing for miR-193b [14]. Similarly, it was shown that silencing of *MIR31HG* significantly inhibited NSCLC cell migration, invasion, and metastasis by attenuated sponging of miR-214 [35]. The various mechanisms of *MIR31HG* suggest that downstream pathways could be involved with other non-coding RNAs, which requires further verification. In this study, similar to the full-length transcript, a series of functional experiments validated the corresponding roles in certain types of BLCA cells. The knock-down of *MIR31HGΔE3* resulted in reduced colony formation ability and cell viability solely in SCaBER cells, suggesting that exon-specific or transcript-specific mechanisms could be a new direction in studying basal-like BLCA. Recently, emerging data have demonstrated that RNA splice variants are associated with drug resistance in cancer. The expression of androgen receptor (AR) splicing variants in castration-resistant prostate cancer (CRPC) samples increased significantly. The most common AR splicing variants are AR-V7 and ARv567es [46, 47]. These variants are important factors for insensitive to AR antagonists of CRPC patients. Furthermore, splice variants in V600E BRAF-mutant-positive malignant melanoma patients were shown to be associated with vemurafenib resistance, indicating that aberrant splicing could be a novel mechanism of acquired resistance [48]. Furthermore, interference of the pre-mRNA splicing modulators sufficiently inhibited formation of these splice variants, suggesting that splice variant-specific siRNAs may be proposed as a therapeutic strategy to overcome drug resistance [49].

For basal or squamous-like bladder cancer, molecular signatures were found based on clustering RNA-seq data, including *EGFR* [34]. *EGFR* is overexpressed in up to 74% of bladder cancer tissue specimens, and is amplified in squamous cell carcinomas (SCC) of the bladder [50]. To further investigate the potential relationship between *MIR31HG* and *EGFR*, a computational method called lncPro was applied to predict the associations within. All the nine isoforms of EGFR, as well as PI3K and HER2 protein, were predicted as interactive with *MIR31HG*. The positive interaction score suggested that *MIR31HG* might be involved in the EGFR/PI3K/AKT signaling pathway. Furthermore, expression of *EGFR* was detected in three *MIR31HG*-knockdown BLCA cell lines. In SCaBER cells, which shows the basal / squamous signature, expression of *EGFR* was reversely correlated with *MIR31HG*. According to another study on lung cancer, by knocking down *MIR31HG*, reversal of gefitinib resistance was found by regulation of the EGFR/PI3K/AKT signaling pathway [51]. Taken together, these findings suggested that *MIR31HG* might potentially correlate with the EGFR pathway.

## Conclusions

In summary, our data show that *MIR31HG* was highly expressed in basal subtype cells and tissues of MIBC. *MIR31HG* and its splice variants (*MIR31HGΔE1* and *MIR31HGΔE3*) could regulate proliferation and migration of corresponding BLCA cells. Furthermore, in different MIBC cohorts, *MIR31HG* and its splice variants were associated with survival of different subgroups of patients. Our research provides new insights into studies for the molecular classification of MIBC.

## Supplementary Information

**Additional file 1: Fig. S1.** Expression of *MIR31HG* in BLCA tissue samples and cell lines based on in silico data. (A) Expression of *MIR31HG* was higher in basal (median expression 7.10 with range of 0 to 9.96) subtype than in luminal (median expression 5.21 with range of 0 to 10.16) and infiltrated (median expression 4.99 with range of 0 to 9.16) subtypes in patients of the TCGA cohort. (B) No significant difference of MIR31HG expression was found between lymph node metastasis negative and positive groups. (C) RNA-seq data from the Cancer Cell Line Encyclopedia showed expression levels in TPM (transcripts per million) for *MIR31HG* in 25 BLCA cell lines. **Fig. S2.** Kaplan-Meier plot of the TCGA cohort of OS and DFS associated with *MIR31HG* risk stratification. The group with high or low expression of *MIR31HG* showed no significant correlation with OS (A, median survival, 16 vs. 15 months, *p* = 0.9638) and DFS (B, median survival, 15 vs. 17 months, *p* = 0.4175) in the whole TCGA cohort. The numbers below the figures showed the number of patients at risk in each group. **Fig. S3.** Kaplan-Meier plot of the TCGA cohort with basal subtype of overall survival associated with *MIR31HG* Exon1–2 (Junction 3) risk stratification. (A) The group with high Junction 3 expression showed worse OS than the group with low expression (median survival, 17 vs. 14 months, *p* = 0.0298). (B) No significant difference was observed in DFS between the group with high and low Junction 3 expression (median survival, 17 vs. 15 months, *p* = 0.5670). The numbers below the figures showed the number of patients at risk in each group. **Fig. S4.** Kaplan-Meier plot of the Mannheim cohort with basal subtype of OS and DFS associated with *MIR31HG* and its splice variants risk stratification. No significant correlation was found with OS (A, median survival 18 vs. 20 months) and DFS (B, median survival 17 vs. 15 months) in the group with full-length transcript of *MIR31HG*. No significant correlation was found with OS (C, median survival 15 vs. 18 months) and DFS (D, median survival 29 vs. 17 months) in the group with *MIR31HGΔE1*. The group with high *MIR31HGΔE3* expression showed a worse OS (E, median survival 13 vs. 22 months) compared to the group with low expression, no significant correlation was found with DFS (F, median survival 15 vs. 36 months). The numbers below the figures showed the number of patients at risk in each group. **Table S1.** siRNAs used in this study. **Table S2.** Primers and probes used in this study.

## Data Availability

The data and materials of this study are available from the corresponding author for reasonable requests.

## Abbreviations

BLCA: Bladder urothelial carcinoma
DFS: Disease-free survival
EGFR: Epidermal growth factor receptor
FFPE: Formalin fixed paraffin embedded
lncRNA: Long non-coding RNA
MIR31HG: miR-31 host gene
MIBC: Muscle invasive bladder cancer
OS: Overall survival
siRNA: small interfering RNA
TCGA: The Cancer Genome Atlas
TPM: Transcripts per million
