# Supplementary material for: Long noncoding RNA MIR31HG and its splice variants regulate proliferation and migration: prognostic implications for muscle invasive bladder cancer
Source: J Exp Clin Cancer Res. 2020 Dec 17;39:288. doi: 10.1186/s13046-020-01795-5 (PMC7745499; doi:10.1186/s13046-020-01795-5)
Supplement: Supplementary file 1 — Additional file 1: Fig. S1. Expression of MIR31HG in BLCA tissue samples and cell lines based on in silico data. (A) Expression of MIR31HG was higher in basal (median expression 7.10 with range of 0 to 9.96) subtype than in luminal (median expression 5.21 with range of 0 to 10.16) and infiltrated (median expression 4.99 with range of 0 to 9.16) subtypes in patients of the TCGA cohort. (B) No significant difference of MIR31HG expression was found between lymph node metastasis negative and positive groups. (C) RNA-seq data from the Cancer Cell Line Encyclopedia showed expression levels in TPM (transcripts per million) for MIR31HG in 25 BLCA cell lines. Fig. S2. Kaplan-Meier plot of the TCGA cohort of OS and DFS associated with MIR31HG risk stratification. The group with high or low expression of MIR31HG showed no significant correlation with OS (A, median survival, 16 vs. 15 months, p = 0.9638) and DFS (B, median survival, 15 vs. 17 months, p = 0.4175) in the whole TCGA cohort. The numbers below the figures showed the number of patients at risk in each group. Fig. S3. Kaplan-Meier plot of the TCGA cohort with basal subtype of overall survival associated with MIR31HG Exon1–2 (Junction 3) risk stratification. (A) The group with high Junction 3 expression showed worse OS than the group with low expression (median survival, 17 vs. 14 months, p = 0.0298). (B) No significant difference was observed in DFS between the group with high and low Junction 3 expression (median survival, 17 vs. 15 months, p = 0.5670). The numbers below the figures showed the number of patients at risk in each group. Fig. S4. Kaplan-Meier plot of the Mannheim cohort with basal subtype of OS and DFS associated with MIR31HG and its splice variants risk stratification. No significant correlation was found with OS (A, median survival 18 vs. 20 months) and DFS (B, median survival 17 vs. 15 months) in the group with full-length transcript of MIR31HG. No significant correlation was found with OS (C, median [file 13046_2020_1795_MOESM1_ESM.zip › Supplementary Material_3.docx]

Long noncoding RNA *MIR31HG* and its splice variants regulate proliferation and migration: prognostic implications for muscle invasive bladder cancer

Sheng Wu^1,2^, Katja Nitschke^1^, Thomas Stefan Worst^1^, Alexander Fierek^1^, Cleo-Aron Weis^3^, Markus Eckstein^4^, Stefan Porubsky^3^, Maximilian Kriegmair^1^, Philipp Erben^1^***

**

**

**Fig. S1.** Expression of *MIR31HG* in BLCA tissue samples and cell lines based on *in silico* data. (A) Expression of *MIR31HG* was higher in basal (median expression 7.10 with range of 0 to 9.96) subtype than in luminal (median expression 5.21 with range of 0 to 10.16) and infiltrated (median expression 4.99 with range of 0 to 9.16) subtypes in patients of the TCGA cohort. (B) No significant difference of MIR31HG expression was found between lymph node metastasis negative and positive groups. (C) RNA-seq data from the Cancer Cell Line Encyclopedia showed expression levels in TPM (transcripts per million) for *MIR31HG* in 25 BLCA cell lines.





**Fig. S2.** Kaplan-Meier plot of the TCGA cohort of OS and DFS associated with *MIR31HG* risk stratification. The group with high or low expression of *MIR31HG* showed no significant correlation with OS (A, median survival, 16 vs. 15 months, *p* = 0.9638) and DFS (B, median survival, 15 vs. 17 months, *p* = 0.4175) in the whole TCGA cohort. The numbers below the figures showed the number of patients at risk in each group.

**

**

**Fig. S3.** Kaplan-Meier plot of the TCGA cohort with basal subtype of overall survival associated with *MIR31HG* Exon1-2 (Junction 3) risk stratification. (A) The group with high Junction 3 expression showed worse OS than the group with low expression (median survival, 17 vs. 14 months, *p* = 0.0298). (B) No significant difference was observed in DFS between the group with high and low Junction 3 expression (median survival, 17 vs. 15 months, *p* = 0.5670). The numbers below the figures showed the number of patients at risk in each group.





**Fig. S4.** Kaplan-Meier plot of the Mannheim cohort with basal subtype of OS and DFS associated with *MIR31HG* and its splice variants risk stratification. No significant correlation was found with OS (A, median survival 18 vs. 20 months) and DFS (B, median survival 17 vs. 15 months) in the group with full-length transcript of *MIR31HG*. No significant correlation was found with OS (C, median survival 15 vs. 18 months) and DFS (D, median survival 29 vs. 17 months) in the group with *MIR31HGΔE1*. The group with high *MIR31HGΔE3* expression showed a worse OS (E, median survival 13 vs. 22 months) compared to the group with low expression, no significant correlation was found with DFS (F, median survival 15 vs. 36 months). The numbers below the figures showed the number of patients at risk in each group.

| **Table S1.** siRNAs used in this study. | |
| --- | --- |
| **Target gene** | **Sequence (5' → 3')** |
| *MIR31HG* | GUUGAUGGUUAUUAGUGAA |
|  | GCGCUUUGUGUGAGAAGUU |
|  | AGGUUAUAUCCUAGAGAUC |
|  | CAUAGAACCUUGGAUCCUA |
| *MIR31HGΔE1* | CCCAGGAGGAGCUUGGUUUCUGGUU |
| *MIR31HGΔE3* | GAGGAUUCAUUCCAAGGUAGAGAUU |

| **Table S2.** Primers and probes used in this study. | | | |
| --- | --- | --- | --- |
| **Gene** | **Forward Primer Sequence (5' → 3')** | **Reverse Primer Sequence (5' → 3')** | **Probe Sequence (5' label→ 3' label)** |
| *Calm2* | GAGCGAGCTGAGTGGTTGTG | AGTCAGTTGGTCAGCCATGCT | VIC-TCGCGTCTCGGAAACCGGTAGC-BHQ1 |
| *EGFR* | CGCAAGTGTAAGAAGTGCGAA | CGTAGCATTTATGGAGAGTGAGTCT | FAM-CCTTGCCGCAAAGTGTGTAACGGAAT-BHQ2 |
| *GUS* | GAAAATAYRTGGTTGGAGAGCTCATT | CCGAGTGAAGATCCCCTTTTTA | VIC-CCAGCACTCTCGTCGGTGACTGTTCA-BHQ1 |
| *KRT5* | CGCCACTTACCGCAAGCT | ACAGAGATGTTGACTGGTCCAACTC | FAM-TGGAGGGCGAGGAATGCAGACTCA-BBQ |
| *KRT20* | GCGACTACAGTGCATATTACAGACAA | CACACCGAGCATTTTGCAGTT | FAM-TTGAAGAGCTGCGAAGTCAGATTAAGGATGCT-BBQ |
| *MIR31HG* | CTCTGGAGGACAGAGGATTCATTCC | TGGGAGGGTGGTCTGAAACTG | FAM-GGGTCTGCTTGTATTCAATGACTGGTCTACGTGGG-BHQ1 |
| *MIR31HGΔE1* | GCCTCCCAGGAGGAGC | CCAAACTCTGGAGGACAGAGGATTC | FAM-CAGGTTTCTGGTCCTCATACCGTGTGGT-BHQ1 |
| *MIR31HGΔE3* | CTCTGGAGGACAGAGGATTCATTCC | GGAACACCTGGAGACCTGCT | FAM-GGTAGAGATGGATTCCTGGAAATACCTCCTCAAGGCC-BHQ1 |
